# Age dependent vestibulo-ocular reflex gain in video head impulse test among normal Indians

**DOI:** 10.6026/973206300200520

**Published:** 2024-05-31

**Authors:** Saraswathi Avula, Saran Kumar, Senthi Vadivu, Mohan Kameswaran

**Affiliations:** 1 Department of ENT, GITAM Institute of Medical sciences and Research, GITAM Deemed to be University, Visakhapatnam, Andhra Pradesh, India; 2 Madras ENT Research Foundation, Chennai, India

**Keywords:** Vestibulo-ocular reflex, video head iimpulse test, vertigo, vestibular loss

## Abstract

Determination of the normative data of Vestibulo-Ocular Reflex gain using VHIT of all three semicircular canals (anterior, posterior, horizontal) on both sides in different age groups is of interest. This is an observational study comprised of 10 healthy individuals in each decade from less than 10 years to 80 years of both sexes making a total of 80. The study was done using the equipment SYNAPSYS VHIT ULMER with Software EVOLUTION 3.0. Mean VOR gain of each decade for all the semicircular canals is calculated and they are compared using ANOVA (Analysis of variance). In our study, percentage of patients with overt saccades is nil. Hence, in our study, the occurrence of covert saccades was insignificant as compared to the above studies. Age dependent VOR gain in normal individuals did not have any significance in our study of 80 patients performed by video head impulse test. VOR gain in our study is not affected by age. Normative data for different age groups obtained compared among different age groups showing no significant difference in Mean VOR gain. The normative values of VOR gain can be compared to patients with dizziness thus helping in determining any vestibular loss.

## Background:

Vertigo, often known as dizziness, is an uncomfortable disruption of one's sense of space or an illusion of movement, either of the body or of the environment. About 10-20% of patients examined by neurologists and otolaryngologists have vertigo, dizziness, or disequilibrium [1]. The cerebellum, extrapyramidal system, reticular formation, and cortex are among the nervous system's modulators of the intricate physiology of balance and equilibrium, which incorporates visual, proprioceptive, and vestibular inputs [1]. Vertigo may be caused by a central lesion, peripheral vestibular disease, or a combination of both. There is no one test that can determine the cause of vertigo [1]. A series of tests is required to pinpoint the precise location of the pathology. One of the most crucial first issues in the evaluation of patients with dizziness is whether or not a vestibular impairment is present. The rotational and caloric tests have historically been used to assess the function of the horizontal semicircular canal. The caloric test however is anunphysiological test as the afferent stimulus (hot and cold water or air) is not the physiologic stimulus to the labyrinth [2]. Low frequency stimulus is used for caloric tests (0.003Hz) and rotatory chair test (0.01 to 0.64 Hz). Caloric testing generates a very low-frequency stimulation of the Horizontal SCCs which is estimated to be equivalent to head movements with a frequency of approximately 0.003Hz.A newly developed test, Head Impulse Test in the last decade is based on the vestibulo-ocular reflex[2]. "In the simplest form, the head impulse test provides fast, reliable, bedside screening of semicircular canal (SCC) function with no equipment needed [2]." Halmagyi and Curthoys reported HIT not only demonstrates "catch-up saccades" and other abnormalities in patients with impaired VOR function, it allows a three-dimensional analysis with respect to the three pairs of SCCs and HIT complements traditional vestibular tests. Vestibulo -ocular reflex is the reflex mechanism that carries out the function of gaze stabilization which is one of the most important functions of balance system [2]. It helps to stabilize the image on fovea when the visual target moves but the head is steady, when the head moves but visual target is steady and when both the head as well as visual target are moving together. This test has the advantage of testing the labyrinth by a physiologically relevant stimulus (high velocity head movement) at physiologically relevant velocities and also allows the evaluation of such function in the horizontal and vertical planes [2].VHIT provides a quick and objective measure of the vestibular ocular reflex (VOR) in response to head movements in the natural range of daily motions. The Video head impulse (VHIT) is more sophisticated equipment with the advantage of objective evaluation and to assess covert saccades better [2]. Therefore, it is of interest to evaluate age dependent vestibulo-ocular reflex gain in video head impulse test among normal Indians individuals.

## Materials and Methods:

## Study Design:

This is an observational study conducted at Madras ENT Research Foundation (p) Ltd, Chennai.

## Study Duration:

This study was conducted for a period of one year (July 2016- June2017)

## Place of study:

This study was conducted at vertigo clinic of Madras ENT Research Foundation (p) Ltd, Chennai.

## Study Population:

Study population includes 10 individuals in each decade from less than 10years to 80years of both sexes making a total of 80.

## Inclusion criteria:

[1] Normal healthy asymptomatic individual with normal vestibular tests clinically.

[2] Age ranges from less than 10 years to 80 years of both sexes.

## Exclusion criteria:

[1] Individuals having any ear related complaints, any dysfunction of ocular movements, corneal opacities and cataract.

[2] Individuals with severe cervical spondylosis, cervical injury or surgery of cervical spine.

[3] Individuals who underwent any ear or eye surgeries.

[4] Patients with central nervous system symptomatology

## Ethical clearance :

The proposal was approved by the institutional review board at MERF in August 2016.

## Equipment:

The study was done using the equipment SYNAPSYS VHIT ULMER with Software EVOLUTION 3.0.The VHIT Ulmer is designed to assess the vestibular-ocular-reflex (VOR) by measuring, recording, displaying and analyzing eye and head movements. The system, developed jointly by Doctor Erik Ulmer and the company SYNAPSYS, consists of a camera assembled on a monopod connected to a computer via a USB port. The VHIT Ulmer is equipped with an ultra-sensitive camera that films the patient's face from a distance of approximately 90 cm. The camera's built-in infra-red lighting allows obtaining clear and contrasted, even in the case of rapid head movements. The equipment is composed of the following:

[1] The motorized VHIT camera head

[2] An adjustable supporting monopod

[3] A type AB USB cable of 3 meters

[4] A medical grade 13.2V power supply with cables

[5] Synapsys VHIT Ulmer software provided on CD.

The equipment also comprises of Built-in gyroscopes which ensure accurate VOR gain measurement and provide instant feedback on proper head impulse maneuver. Built-in calibration lasers where Software features allow describing the VOR results, using video, calculated values, graphs and diagrams like the Canalogram Ulmer. Canalogram Ulmer presents in a single diagram, VOR gain values obtained for each impulse on each semi-circular canal.

## Methodology:

All the patients included in the study were explained about the purpose of the study and were ensured that the information collected from them would be kept confidential and would be used only for academic purpose. Patient information sheet was provided and written informed consent was taken. All test subjects were healthy, independent, community-dwelling individuals with no history of any vestibular disorder. They were recruited from hospital staff and their associates as well as community groups for seniors. At least 10 subjects in each age decade from less than 10years to 80 years without any prior known or reported balance problems were included in the study. After thorough history and clinical examination as per the study proforma:

[1] Hearing was checked with tuning forks in three frequencies.

[2] Other otoneurlogical tests like Romberg's test, unterberger test, head shake test, positional test were carried out.

[3] Subjects are also tested for any spontaneous and gaze induced nystagmus.

[4] Cerebellar function tests were also performed on subjects.

[5] Clinical head impulse test was performed which was normal in all individuals. It was performed by the researcher under the supervision of thesis guide.

## Video Head Impulse Test:

Necessary information regarding the handling and use of the system was obtained from the SYNAPSYS ULMER represented and appropriate training for performing the video head impulse test effectively was undertaken by the researcher and the guide. The patients were instructed to keep eyes wide open and fixate their vision on a target at about 1m distance and also to keep neck relaxed but teeth clenched to avoid movement artefacts. Patient was positioned on a preferably immovable chair. To begin the test, the test is setup for the plane of the impulse i.e. lateral, LARP(Left anterior- Right posterior) or RALP (Right anterior- Left posterior), and the pupil is marked on the computer screen so that it is detected by the camera. Before starting the test the system needs to be calibrated for each patient. The Synapsys Ulmer has an inbuilt calibration system which makes the patient fix the vision at alternate specified target dots at predefined angles. The height of the camera was set to match the patient's eyes, using the adjustable stand. The camera position was fine-tuned by operating the camera -head motors via the VHIT Ulmer II software. The patients' eyes must be centered horizontally in the picture and centered vertically in Yellow Square on the video. A correct startup positioning of VHIT ensures to make impulses on each canal recorded without any further adjustment. For optimum pupil detection, it is essential to adjust the inter-pupillary distance. Based on the ability of the testing person to carry out the head movements, the acceleration thresholds can be modified to a minimum of 15000/s2. The camera was placed on floor stand. Fixation dots were placed on a horizontal line point of sight above the camera so that they remain visible for the patient. The examiner stands behind the patient and moves the head in sudden unpredictable jerks horizontally (for lateral canals) or vertically (for the vertical canals) while the subject keeps eyes focused on a visual target marked on a wall. The infrared camera records the head movements as well as eye movements and the pictures taken every 10 milli -seconds are analyzed for locating the position of head and that of eyes. Then position of head and eye movement are graphically plotted. The test was performed till 5-7 valid impulses were obtained for each canal. There are six distinct types of head impulse

[1] Horizontal movement to the right, tests the right lateral canal,

[2] Horizontal movement to the left, tests the left lateral canal,

[3] Head turned 35° to 45° towards the right, forward impulse in the sagittal plane, tests the left anteriorcanal,

[4] Head turned 35° to 45° towards the right, backward impulse in the sagittal plane, tests the right posteriorcanal,

[5] Head turned 35° to 45° towards the left, forward impulse in the sagittal plane, tests the right anterior canal,

[6] Head turned 35° to 45° towards the left, backward impulse in the sagittal plane, tests the left posteriorcanal.

Vestibulo ocular reflex gain is represented in form of a canalogram. Canalogram ULMER is made of 6 branches, divided in 2 half-branches: White half-branches for VOR gains and Grey half-branches for apparent gain after early saccade if any. At the end of branches, final result of VOR gain will be shown as well as the number of valid collected impulses for each canal. The colored area at the end of each branch can wear 3 different colors:

Grey: Not enough valid impulses for this canal to compare results with normative limits. 5valid impulses are required.

Green: Mean VOR gain of this canal is within normative limits.

Red: Mean VOR gain of this canal is outside normative limits.

VOR gain is calculated by VHIT software based on head velocity curve and eye velocity curves. The calculation process can be broken in 3 steps:

[1] Head velocity and eye velocity curves region selection in the time interval {t0; t1}

[2] Curves are fitted using least squares method with 2 varying parameters : gain et latency

[3] VOR gain is found when error between curves reaches its minimum.

VHIT was administered to the individuals in the study group. The test was done by one person in our institution laboratory. The Average VOR gain of each individual obtained from ulmer canalogram was entered in data chart. There are 8 study groups of each decade (less than 10 yrs - 80 yrs).Mean VOR gain of each decade for all the semicircular canals is calculated and they are compared using ANOVA (Analysis of variance).

## Results:

In our study comprising of 80 healthy individuals, 42 (52.5%) were males and 38 (47.5%) were female patients. Table 1 depicts the Average VOR gain of Right Anterior semicircular canal of each individual from 0-80 years respectively. The Mean VOR gains for Right Anterior SCC are calculated and are depicted in the Table 2 respectively. There was no mean significant (p- 0.261) difference in VOR gain of RA SCC in different age groups (Table 3). I.e. the mean VOR gain of RA SCC found to be equal in almost all the age groups. One-way analysis for VOR Gain of Right Anterior Semicircular Canal showed insignificant results. Table 4 depicts the Average VOR gain of Left Anterior semicircular canal of each individual from 0-80 years respectively. The Mean VOR gains for Left Anterior SCC are calculated and are depicted in the Table 5 respectively. Table 6 showed that there was no mean significant (p- 0.263) difference in VOR gain of LA SCC in different age groups. The mean VOR gain of LA SCC found to be equal in almost all the age groups. Table 7 depicts the Average VOR gain of Right Lateral semicircular canal of each individual from 0-80 years respectively. The Mean VOR gain for Right lateral SCC is calculated and are depicted in the Table 8 respectively. Table 8 showed that there was no mean significant (p- 0.147) difference in VOR gain of RL SCC in different age groups. The mean VOR gain of RL SCC found to be equal in almost all the age groups and the results were insignificant (Table 9). Table 10 depicts the Average VOR gain of Left Lateral semicircular canal of each individual from 0-80 years respectively. The Mean VOR gains for Left lateral SCC is calculated and is depicted in the Table 11 respectively. Table 12 showed that there was no mean significant (p = 0.830) difference in VOR gain of LL SCC in different age groups. The mean VOR gain of LL SCC found to be equal in almost all the age groups. Table 13 depicts the Average VOR gain of Right Posterior semicircular canal of each individual from 0-80 years respectively. The mean VOR gain for Right posterior SCC is calculated and are depicted in the Table 14 respectively and the results showed insignificant (Table 15). The average and Mean VOR gain for Left posterior SCC is calculated and depicted in the Table 16 and Table 17 respectively.Table 17 showed that there was no mean significant (p-0.218) difference in VOR gain of LP SCC in different age groups. The mean VOR gain of LP SCC found to be equal in almost all the age groups. We have found that there is no significant difference of Mean VOR gain of all 6 semicircular canals among different age groups (Table 18). The mean VOR gain and SD for each canal are calculated for all the individuals (80). Mean VOR for each canal is shown in table no 20. Hence the normative data for 6 semicircular canals are shown in Table 19. All the statistical analysis was done by using SPSS trial version-16. Quantitative variables were expressed as Mean ± 2SD. Analysis of variance (ANOVA) was used for comparison of all the group means. The statistical analysis at p<0.05 is considered as statistically significant.

## Discussion:

In our study of 80 patients, we completed oto-neurological tests, tuning fork tests, and clinical head impulse test followed by video head impulse test. The video head impulse test report as described earlier provides us with vast and accurate information regarding the individual semicircular canals, namely the VOR gain. The findings are elaborated in the form of tables and line diagrams. VOR gain is calculated automatically for each semicircular canal and displayed for each proper impulse. In our study, we have considered two parameters, namely, VOR gain and its comparison in different age groups for analysis. The cut off value of VOR gain determined by ULMER evolution software of Vhit Synapsys equipment of our study are 0.8 - 1.2 in lateral canals and 0.7- 1.2 for vertical canals. A Study published with 20 healthy subjects using EyeSeeCamvHIT system set the normal VOR gain as0.79 or greater [3]. Bell *et al.* redefined the VOR gain normative upper limit 1.21 and lower limits 0.83 of two lateral semicircular canals combined based on 30 asymptomatic healthy subjects using the GN-Otometrics vHIT system [4]. Nicolas Perez-Fernandez, PalomaEza-Nunez performed VHIT in 623 patients and found 36 patients with normal gains and refixation saccades. Among them, only overt saccades were found in 24 (67%) patients, and covert and overt saccades were found in 12 (33%) [5]. However in our study, percentage of patients with overt saccades is nil. Hence, in our study, the occurrence of covert saccades was insignificant as compared to the above studies. Age dependent VOR gain in normal individuals did not have any significance in our study of 80 patients performed by video head impulse test. Normative data for horizontal canal VOR gain using the video-oculography device was obtained by Mossman, Purdie and Schneider (2012) [6]. 60 normal subjects with an age range of 20 to 80 years were tested with vHIT. They reported a very minimal reduction in VOR gain with age and non-physiologically high gain when subjects were tested in an overly predictable pattern. Their result revealed VOR gain measurements that were very similar to sclera search coil VOR gain studies [6]. Blowdow *et al.* [3] showed an average gain of 0.96/0.97 for the healthy controls, similar to findings from Mossman's group. In our study Synapsys equipment does not display the head velocity values, VOR gain is automatically calculated by the software. VOR gain in our study is not affected by age. Leigh A. McGarvie *et al.* done a study on 10 healthy subjects in decade age bands: 10-19, 20-29, 30-39, 40-49, 50-59, 60-69, 70-79, 80-89 showed Vestibulo-ocular reflex gain decreased at high head velocities, but was largely unaffected by age into the 80- to 89-year age group [7]. Sample size of our study was 80 normal healthy individuals without any balance disorders. Sample size can be bigger as done by Nicolas Perez-Fernandez, PalomaEza-Nunez who performed VHIT in 623 patients [5]. In our study the equipment is not provided with goggles, so problem of slippage, artifacts are avoided, where as in GN-Otometrics, Eye See Cam, lightweight video-oculography camera integrated to goggle that is tightly fixed to the head. During this study period, we also performed vHIT test as an initial investigation for patients presenting to the emergency with acute vertigo, and VHIT was found useful for excluding a peripheral lesion and a neurological workup could be asked for. However, these patients were not included in the study as they did not fit into the predefined inclusion criteria.

## Limitations of the study:

In our study, the actual head velocity was not obtained for each stimulus and hence VOR gain with varying head velocity is not determined. As many studies show VOR gain increase with head velocity. Our study population is smaller and a more elaborative study will give precision in values. The study population is limited to few regions of the state and hence the applicability to wider region of different ethnic groups may be a concern. Patients with cervical spondylosis and ophthalmic disorders preventing fixation of vision, cannot be tested with VHIT.

## Strength of the study:

All age groups could be tested without any difficulty. This study is first of its kind (obtaining normative data) in our country.

## Conclusion:

Data shows that age dependent VOR gain did not have any significant impact on normal individuals performed by video head impulse test. In addition, the study also found that there was no significant difference of Mean VOR gain of all 6 semicircular canals among different age groups the normative values of VOR gain can be compared to patients with dizziness thus helping in determining any vestibular loss.

## Figures and Tables

**Table 1:** Average VOR gain of right anterior semicircular canal

**Patient**	**Age in years**						
	**0 to 10**	**11 to 20**	**21 to 30**	**31 to 40**	**41 to 50**	**51 to 60**	**61 to 70**	**71 to 80**
1	0.97	0.74	1.13	0.98	0.94	0.99	0.9	0.94
2	1.02	1.02	1.06	1.11	1.05	0.97	0.97	0.96
3	0.93	1.02	0.94	1	0.99	1.14	1.13	0.74
4	1.11	1.03	0.8	0.94	1.08	1.03	0.99	0.94
5	0.95	0.84	0.93	1	1.01	1.06	1.04	0.9
6	0.83	0.99	0.98	1.02	1.06	1.01	1	0.96
7	1.02	0.95	0.92	0.98	1.03	1.01	1.1	1.12
8	0.98	1.02	1.03	0.99	1.02	0.99	1.08	1
9	1.02	0.96	0.99	1.1	0.97	1.01	0.97	0.96
10	0.98	1.02	1.12	0.92	1.14	0.99	0.98	1.02

**Table 2:** Mean VOR gain of right anterior semicircular canal

**AGE GROUP**	**N**	**Mean**	**Std. Deviation**	**Minimum**	**Maximum**	**P VALUE**
0-10	10	0.981	0.07279	0.83	1.11	0.261
20-Nov	10	0.959	0.09608	0.74	1.03	
21-30	10	0.99	0.10011	0.8	1.13	
31-40	10	1.004	0.06077	0.92	1.11	
41-50	10	1.029	0.05744	0.94	1.14	
51-60	10	1.02	0.04899	0.97	1.14	
61-70	10	1.016	0.07043	0.9	1.13	
71-80	10	0.954	0.09617	0.74	1.12	
Total	80	0.9941	0.07862	0.74	1.14	

**Table 3:** One -ANOVA analysis for VOR gain of right anterior semicircular canal of groups

**ANOVA**
**RA**	**Sum of Squares**	**Df**	**Mean Square**	**F**	**Sig.**
Between Groups	0.055	7	0.008	1.304	0.261
Within Groups	0.433	72	0.006		
Total	0.488	79			

**Table 4:** Average VOR gain of left anterior semicircular canal

**Patient**	**Age in years**							
	**0 to 10**	**11 to 20**	**21 to 30**	**31 to 40**	**41 to 50**	**51 to 60**	**61 to 70**	**71 to 80**
1	0.81	0.84	1.08	1	0.98	1.01	1	0.95
2	0.98	0.95	1.06	0.98	1.01	1	0.95	1
3	1.06	1.01	1.05	1.09	1.01	1.12	1.03	0.97
4	1.06	1.03	0.94	0.98	1.14	0.95	0.94	0.99
5	0.99	0.89	0.98	1	0.99	1	0.94	1
6	0.91	1.01	0.96	0.98	1.04	1.07	1.01	1
7	1.1	0.98	0.8	1.06	1.1	1.05	1.03	1.14
8	0.88	1.06	1.05	1.01	1.03	1.01	1.14	1
9	1.01	0.99	1.03	1.13	1.1	1.05	0.95	0.98
10	1.11	1	1.05	1.13	1.08	1.01	1.02	1

**Table 5:** Mean VOR gain of left anterior semicircular canal

**AGE GROUP**	**N**	**Mean**	**Std. Deviation**	**Minimum**	**Maximum**	**P VALUE**
0-10	10	0.991	0.09871	0.81	1.11	0.263
20-Nov	10	0.976	0.06637	0.84	1.06	
21-30	10	1	0.08433	0.8	1.08	
31-40	10	1.036	0.06132	0.98	1.13	
41-50	10	1.048	0.05391	0.98	1.14	
51-60	10	1.027	0.04692	0.95	1.12	
61-70	10	1.001	0.06154	0.94	1.14	
71-80	10	1.003	0.051	0.95	1.14	
Total	80	1.0102	0.06845	0.8	1.14	

**Table 6:** One-ANOVA analysis for VOR gain of left anterior semicircular canal of groups

**LA**	**Sum of Squares**	**Df**	**Mean Square**	**F**	**Sig.**
Between Groups	0.042	7	0.006	1.301	0.263
Within Groups	0.329	72	0.005		
Total	0.37	79			

**Table 7:** Average VOR gain of right lateral semicircular canal

**Patient**	**Age in years**							
	**0 to 10**	**11 to 20**	**21 to 30**	**31 to 40**	**41 to 50**	**51 to 60**	**61 to 70**	**71 to 80**
1	0.98	1.05	0.92	1.02	1.01	1	0.93	1.08
2	1.1	0.96	0.81	1.01	1.02	0.97	1.02	1.03
3	0.89	0.99	1.07	0.94	1.01	1.09	1.02	0.87
4	0.84	1	1.01	0.96	1.04	0.87	0.99	0.84
5	0.84	0.95	1.02	0.95	1.04	0.93	1.03	0.98
6	0.94	1.03	0.99	1	0.99	1.01	1.03	1.03
7	1.05	0.98	1.03	0.91	1.03	0.92	1.02	0.94
8	0.89	1	0.96	1.01	0.97	1	0.92	0.96
9	0.93	1.01	0.99	0.9	1	0.92	1.02	1.04
10	0.86	0.99	1.07	1.01	0.97	0.89	0.98	1

**Table 8:** Mean VOR gain of right lateral semicircular canal

**AGE GROUP**	**N**	**Mean**	**Std. Deviation**	**Minimum**	**Maximum**	**P VALUE**
0-10	10	0.932	0.08829	0.84	1.1	0.147
20-Nov	10	0.996	0.02989	0.95	1.05	
21-30	10	0.987	0.07732	0.81	1.07	
31-40	10	0.971	0.04483	0.9	1.02	
41-50	10	1.008	0.02573	0.97	1.04	
51-60	10	0.96	0.0665	0.87	1.09	
61-70	10	0.996	0.04088	0.92	1.03	
71-80	10	0.977	0.07646	0.84	1.08	
Total	80	0.9784	0.0621	0.81	1.1	

**Table 9:** One -way ANOVA analysis of averageVOR gain of right lateral semicircular canal

**ANOVA**					
**RL**	**Sum of Squares**	**Df**	**Mean Square**	**F**	**Sig.**
Between Groups	0.041	7	0.006	1.607	0.147
Within Groups	0.264	72	0.004		
Total	0.305	79			

**Table 10:** Average VOR gain of left lateral semicircular canal

**Patient**	**Age in years**							
	**0 to 10**	**11 to 20**	**21 to 30**	**31 to 40**	**41 to 50**	**51 to 60**	**61 to 70**	**71 to 80**
1	0.97	0.95	0.96	0.95	1.03	0.88	0.96	0.99
2	0.97	1.01	0.9	0.98	0.95	1.02	1	0.99
3	1.08	0.99	1	1	1	1.16	1	0.88
4	0.91	1.02	0.96	0.96	0.89	0.88	0.92	0.94
5	0.92	0.95	1.02	0.92	0.99	0.8	0.99	1.01
6	1.17	1.04	0.98	0.99	1	1.01	0.82	0.99
7	1.02	0.94	1.04	0.82	0.98	0.86	1	1
8	1.1	1.05	0.99	1	0.97	0.99	1.01	1.02
9	0.92	0.82	1.02	0.95	0.98	0.99	1	0.99
10	0.92	0.99	1.05	1	1.02	0.98	0.98	0.88

**Table 11:** Average VOR gain of left lateral semicircular canal

**AGE GROUP**	**N**	**Mean**	**Std. Deviation**	**Minimum**	**Maximum**	**P VALUE**
0-10	10	0.998	0.09114	0.91	1.17	0.83
20-Nov	10	0.976	0.0667	0.82	1.05	
21-30	10	0.992	0.04467	0.9	1.05	
31-40	10	0.957	0.05519	0.82	1	
41-50	10	0.981	0.03957	0.89	1.03	
51-60	10	0.957	0.10361	0.8	1.16	
61-70	10	0.968	0.05846	0.82	1.01	
71-80	10	0.969	0.05131	0.88	1.02	
Total	80	0.9748	0.06572	0.8	1.17	

**Table 12:** One-way analysis of VOR gain of left lateral semicircular canal

**ANOVA**
**LL**	**Sum of Squares**	**Df**	**Mean Square**	**F**	**Sig.**
Between Groups	0.016	7	0.002	0.502	0.83
Within Groups	0.325	72	0.005		
Total	0.341	79			

**Table 13:** Average VOR gain of right posterior semicircular canal

**Patient**	**Age in years**							
	**0 to 10**	**11 to 20**	**21 to 30**	**31 to 40**	**41 to 50**	**51 to 60**	**61 to 70**	**71 to 80**
1	0.91	1.07	1.02	1	0.94	1.11	0.88	1.01
2	0.98	0.96	1.02	0.87	0.97	1	0.91	0.92
3	0.87	1	1.09	1.05	0.94	1.11	0.96	0.96
4	0.83	1.02	0.94	0.98	1.04	1	0.91	0.89
5	1.01	0.97	0.86	0.98	1.01	0.93	0.84	0.88
6	0.88	0.98	0.95	1	0.97	1.08	0.83	0.92
7	1.1	1.01	1.04	0.81	1.1	0.96	0.96	1.04
8	0.97	1.01	1.03	0.94	0.96	1.04	1.06	1
9	1.02	0.88	1.01	1.05	1.9	0.89	0.91	0.99
10	0.87	0.97	1.06	1.01	0.96	0.89	0.99	0.98

**Table 14:** Mean VOR gain of right posterior semicircular canal

**AGE GROUP**	**N**	**Mean**	**Std. Deviation**	**Minimum**	**Maximum**	**P VALUE**
0-10	10	0.944	0.0854	0.83	1.1	0.19
20-Nov	10	0.987	0.04945	0.88	1.07	
21-30	10	1.002	0.0673	0.86	1.09	
31-40	10	0.969	0.07666	0.81	1.05	
41-50	10	1.079	0.29278	0.94	1.9	
51-60	10	1.001	0.08359	0.89	1.11	
61-70	10	0.925	0.0698	0.83	1.06	
71-80	10	0.959	0.05405	0.88	1.04	
Total	80	0.9833	0.12536	0.81	1.9	

**Table 15:** One-way analysis of VOR gain of right posterior semicircular canal

**ANOVA**					
**RP**	**Sum of Squares**	**Df**	**Mean Square**	**F**	**Sig.**
Between Groups	0.156	7	0.022	1.475	0.19
Within Groups	1.086	72	0.015		
Total	1.242	79			

**Table 16:** Average VOR gain of left posterior semicircular canal

**Patient**	**Age in years**							
	**0 to 10**	**11 to 20**	**21 to 30**	**31 to 40**	**41 to 50**	**51 to 60**	**61 to 70**	**71 to 80**
1	0.98	0.93	1.01	1	0.88	0.98	0.91	0.98
2	0.87	1.01	1.02	0.84	0.99	0.95	0.89	0.91
3	0.86	0.93	1.03	1.04	0.88	1.04	0.9	0.8
4	0.88	1.02	0.92	0.96	1.08	1.03	0.86	0.92
5	0.85	0.95	0.88	0.98	1.04	0.98	1	0.98
6	0.99	1.08	0.98	0.95	0.9	1.04	0.89	0.91
7	1.11	1.02	1.02	0.79	1.11	0.94	0.9	0.98
8	0.87	0.92	1.02	0.88	1	1.04	1.11	1
9	1.03	0.86	0.98	1.05	1.05	1.04	0.89	0.98
10	0.98	0.97	1.05	1	0.97	0.98	0.99	0.97

**Table 17:** Average VOR gain of left posterior semicircular canal

**AGE GROUP**	**N**	**Mean**	**Std. Deviation**	**Minimum**	**Maximum**	**P VALUE**
0-10	10	0.942	0.08854	0.85	1.11	0.218
20-Nov	10	0.969	0.06402	0.86	1.08	
21-30	10	0.991	0.05322	0.88	1.05	
31-40	10	0.949	0.08608	0.79	1.05	
41-50	10	0.99	0.0826	0.88	1.11	
51-60	10	1.002	0.04022	0.94	1.04	
61-70	10	0.934	0.07648	0.86	1.11	
71-80	10	0.943	0.06019	0.8	1	
Total	80	0.965	0.07204	0.79	1.11	

**Table 18:** One-way analysis of VOR gain of left posterior semicircular canal

**ANOVA**					
**LP**	**Sum of Squares**	**Df**	**Mean Square**	**F**	**Sig.**
Between Groups	0.049	7	0.007	1.401	0.218
Within Groups	0.361	72	0.005		
Total	0.41	79			

**Table 19:** Depicts Mean, SD for each semicircular canal

**SCC**	**VOR Gain in different age groups**								**Mean**	**SD**	**Mean± 2SD**
	**0-10**	**20-Nov**	**21-30**	**31-40**	**41-50**	**51-60**	**61-70**	**71-80**			
RA	0.98	0.96	0.99	1	1.03	1.02	1.02	0.95	0.99	0.04	0.91-1.07
LA	0.99	0.98	1	1.03	1.05	1.03	1	1	1.01	0.02	0.97-1.05
RL	0.93	0.99	0.98	0.97	1.01	0.96	0.99	0.98	0.97	0.02	0.93-1.01
LL	0.99	0.98	0.99	0.95	0.98	0.96	0.97	0.97	0.97	0.01	0.95-0.99
RP	0.94	0.99	1	0.96	1.05	1	0.93	0.96	0.98	0.03	0.92-1.04
LP	0.94	0.97	0.99	0.94	0.99	1	0.93	0.94	0.96	0.02	0.92-1.00
